# Supporting countries to achieve their malaria elimination goals: the WHO E-2020 initiative

**DOI:** 10.1186/s12936-021-03998-3

**Published:** 2021-12-20

**Authors:** Kim A. Lindblade, Hong Li Xiao, Amanda Tiffany, Gawrie Galappaththy, Pedro Alonso, The WHO E-2020 Team, Rabi Abeyasinghe, Kalu Akpaka, Miguel Angel Aragon-Lopez, Ebenezer Sheshi Baba, Anita Bahena, Anderson Chinorumba, Eva Christophel, Camilla Damasceno, Wei Ding, Rainier Escalada, Blanca Escribano, Khoti Gausi, Carolina Gomes, MaryAnne Groepe, Franklin Hernandez, Job Joseph, Elizabeth Juma, James Kelley, Houria Khelifi, Subash Lakhe, Kevin Makadzange, Oscar Mesones-Lapouble, Roberto Montoya, Ahamada Nassuri, Maria-Paz Ade, Enrique Perez-Flores, Risintha Premaratne, Gabriela Rey, Prabhjot Singh, Aida Soto, Abderahmane Kharchi Tfeil, Neena Valecha, Ruan Yao, Ghasem Zamani, Omid Zamani

**Affiliations:** World Health Organization, Geneva, Switzerland

**Keywords:** Malaria, *Plasmodium*, Elimination, Transmission reduction

## Abstract

**Background:**

Malaria causes more than 200 million cases of illness and 400,000 deaths each year across 90 countries. The World Health Organization (WHO) set a goal for 35 countries to eliminate malaria by 2030, with an intermediate milestone of 10 countries by 2020. In 2017, the WHO established the Elimination-2020 (E-2020) initiative to help countries achieve their malaria elimination goals and included 21 countries with the potential to eliminate malaria by 2020.

**Methods:**

Across its three levels of activity (country, region and global), the WHO developed normative and implementation guidance on strategies and activities to eliminate malaria; provided technical support and subnational operational assistance; convened national malaria programme managers at three global meetings to share innovations and best practices; advised countries on strengthening their strategy to prevent re-establishment and preparing for WHO malaria certification; and contributed to maintaining momentum towards elimination through periodic evaluations, monitoring and oversight of progress in the E-2020 countries. Changes in the number of indigenous cases in E-2020 countries between 2016 and 2020 are reported, along with the number of countries that eliminated malaria and received WHO certification.

**Results:**

The median number of indigenous cases in the E-2020 countries declined from 165.5 (interquartile range [IQR] 14.25–563.75) in 2016 to 78 (IQR 0–356) in 2020; 12 (57%) countries reported reductions in indigenous cases over that period, of which 7 (33%) interrupted malaria transmission and maintained a malaria-free status through 2020 and 4 (19%) were certified malaria-free by the WHO. Two countries experienced outbreaks of malaria in 2020 and 2021 attributed, in part, to the COVID-19 pandemic.

**Conclusions:**

Although the E-2020 countries contributed to the achievement of the 2020 global elimination milestone**,** the initiative highlights the difficulties countries face to interrupt malaria transmission, even when numbers of cases are very low. The 2025 global elimination milestone is now approaching, and the lessons learned, experience gained, and updated guidance developed during the E-2020 initiative will help serve the countries seeking to eliminate malaria by 2025.

**Supplementary Information:**

The online version contains supplementary material available at 10.1186/s12936-021-03998-3.

## Background

The *Global technical strategy for malaria 2016–2030* (GTS) of the World Health Organization (WHO) was adopted by the World Health Assembly in 2015 [1, 2]. The GTS maintains the vision of a world free of malaria that was first established in 1955 by the WHO’s Global Malaria Eradication Programme [3]. One of the principles underlying the GTS is that eradication will be achieved through individual country efforts to eliminate malaria within their borders. The GTS recognizes that all countries, irrespective of their current malaria burden, can take steps to accelerate efforts towards elimination through an iterative process of analysis and implementation of intervention packages appropriately tailored to the subnational context. Although countries will follow different trajectories to arrive at elimination depending on their institutional capacity, the strength of their health system, the intensity of malaria transmission in their territory and other social demographic, political and economic realities, the GTS asserts that progress can, and should, be continuous.

One of the three pillars of the GTS calls for all malaria-endemic countries to “accelerate efforts towards elimination and attainment of malaria-free status” [1]. In settings where transmission is very low, nearing elimination, countries are encouraged to intensify efforts to interrupt onward transmission of new infections: in addition to prevention, diagnosis and treatment as part of universal health coverage, the last stages before elimination require an intensive case- and focus-based surveillance and response programme and, possibly, use of medicines and other innovative solutions to reduce the reservoir of infections and prevent transmission from imported cases.

To monitor global progress towards elimination, one of the four goals set by the GTS is for 35 of the 90 countries that were endemic for malaria in 2015 to eliminate malaria by 2030. Milestones have been established at each 5-year mark of the strategy to track progress (Table 1). In 2017, the WHO’s Global Malaria Programme (GMP) established the Elimination 2020 (E-2020) initiative to help countries achieve their individual elimination goals and, as a result, contribute to the 2020 GTS elimination milestone. The E-2020 initiative sought to provide increased visibility, both globally and domestically, to countries’ efforts to eliminate malaria; specialized technical assistance to identify and resolve technical and operational bottlenecks; opportunities for the exchange of innovative approaches and lessons learned between countries from different regions; guidance to accelerate elimination and ease the process of certification; and support to the development of robust programmes to prevent re-establishment of transmission.

**Table 1:** Milestones and targets for the *Global Technical Strategy for Malaria 2016–2030 with the elimination target highlighted*

Goals	Milestones		Targets
	2020	2025	2030
Reduce malaria mortality rates globally compared with 2015	At least 40%	At least 75%	At least 90%
Reduce malaria case incidence globally compared with 2015	At least 40%	At least 75%	At least 90%
Eliminate malaria from countries in which malaria was transmitted in 2015^a^	At least 10 countries	At least 20 countries	At least 35 countries
Prevent re-establishment of malaria in all countries that are malaria-free	Re-establishment prevented	Re-establishment prevented	Re-establishment prevented

^a^Elimination is considered achieved when more than 3 years (i.e., 36 months) have passed with zero indigenous malaria cases reported. Countries are counted as malaria-endemic until they have completed 3 years without indigenous malaria cases. For a country to be included in the achievement of the elimination milestone, it must have interrupted transmission (achieved at least 1 year of zero indigenous cases) and maintained that status through the milestone year. Countries are not officially considered malaria-free until they receive WHO certification

This paper describes the design and implementation of the E-2020 initiative and the programmatic lessons learned that can be used in future to assist countries in reaching their elimination goals. In addition, the paper reports on changes in the number of indigenous malaria cases in the E-2020 countries over the period of the initiative and the number of countries that achieved the WHO certification. Finally, the impact of the COVID-19 pandemic on countries’ abilities to maintain essential health services and their elimination programmes is described.

## Methods

### Selection of the E-2020 countries

In 2016, the WHO identified 21 countries that had a declared malaria elimination goal and were judged to have the potential to achieve elimination by 2020 [4]. The countries included in the E-2020 initiative were: Belize, Costa Rica, Ecuador, El Salvador, Mexico, Paraguay, Suriname (WHO Region of the Americas); China, Malaysia, Republic of Korea (WHO Region of the Western Pacific); Iran (Islamic Republic of), Saudi Arabia (WHO Region of the Eastern Mediterranean); Algeria, Botswana, Cabo Verde, Comoros, Eswatini, South Africa (WHO Region of Africa); and Bhutan, Nepal, Timor-Leste (WHO Region of Southeast Asia).After countries had been selected, the national malaria programmes of Paraguay and Algeria finalized classification of several cases: Paraguay’s last indigenous malaria case was determined to have occurred in 2011 and Algeria’s in 2013. As a result, both countries reported zero indigenous malaria cases in 2016, the baseline year for the E-2020 initiative, which launched in 2017.

### Strategic approach and guiding principles

The strategic approach to the E-2020 initiative was based on the WHO’s key mandates and comparative advantages to other partners working on malaria. First, the WHO is directed by its 194 member-states to provide technical support to countries to achieve improved health outcomes. The organization maintains country offices in 150 nations (including all but 2 of the 90 malaria-endemic countries in 2015) and has six regional offices. Second, the WHO is charged with developing evidence-based, normative guidance that includes recommendations for policies that will help countries achieve their health goals and guidance to assist countries to translate policy recommendations into programmatic strategies and activities. Third, the WHO is the organization mandated by the World Health Assembly to certify countries as malaria-free [5]. As certification requires verification of a country’s malaria-free status as well as proof that the country is able to prevent re-establishment of disease, the WHO plays a fundamental role in assisting countries to establish the systems needed both to achieve elimination and to document the processes and activities that led to achieving that status, developing an effective strategy and program to prevent re-establishment and ensuring countries’ readiness for certification.

Based on the WHO’s responsibilities within malaria elimination, the E-2020 initiative included activities grouped in five strategic areas:

1. Guidance, tools and trainings to assess and strengthen elimination programmes and strategies to prevent re-establishment;
2. Specialized technical and operational assistance to resolve bottlenecks and improve levels of implementation;
3. Networking of national malaria programmes (NMPs) to share innovations and experiences;
4. Preparation for certification to strengthen the programme to prevent re-establishment;
5. Monitoring and oversight to maintain momentum towards elimination and provide course corrections when needed.

The guiding principles under which the initiative operated were the following:

- Elimination is a country-owned and country-driven process.
- NMPs are often innovative in their strategies and have experiences and best practices to share that can benefit other countries in both their own and other regions of the world.
- The process of certification of malaria elimination by the WHO should be more than a stamp of approval but also add value to the country’s prospects for maintaining a malaria-free status.

### “One WHO” to support countries

The WHO operates through three levels of the organization: headquarters, regional and country offices. At country level, national and international staff provide direct support to NMPs to implement malaria strategies and activities. At the regional level, the WHO provides technical support to countries through one or more malaria advisers. At headquarters level in Geneva, Switzerland, the Global Malaria Programme (GMP) develops global normative guidance and provides specialized technical assistance to countries.

With the start of the E-2020 initiative, the WHO added an additional international staff member to each of the five regional offices with malaria-endemic countries (the European region achieved malaria elimination in 2015 and was not included) to provide targeted support to eliminating countries with a focus on the E-2020 initiative. Within the GMP, an Elimination Unit was established in 2016 with three technical staff to oversee the E-2020 initiative and other elimination-related activities.

Throughout the E-2020 initiative, a core principle of the effort was collaboration across the three levels of the WHO to support countries more efficiently and effectively. Although roles and responsibilities varied over time and by region, the initiative was managed by the GMP Elimination Unit with country-specific activities planned and managed by NMPs with support from the WHO country and regional offices. Within each region, the regional elimination focal point organized regional meetings of programme managers, regional progress reviews and cross-border initiatives, in addition to providing technical support to countries and the STOP-malaria consultants. Development of guidance and tools was overseen by the GMP Elimination Unit with significant input from regional and country offices, who often adapted the guidance and tools to better fit their regional context.

### Independent recommendations and oversight

At the start of the E-2020 initiative, the WHO recognized the need for an independent committee comprised of malaria and public health experts that could provide an objective, external and transparent overview of progress towards elimination goals. The Malaria Elimination Oversight Committee (MEOC), composed of 10 members, was established in 2018 to evaluate national and regional progress towards malaria elimination; determine need for corrective actions; identify risks to malaria elimination; identify gaps in policies or guidance for malaria elimination; question the status quo and confront difficult issues [6].

The Malaria Elimination Certification Panel (MECP) was convened in 2017 with 11 malaria experts to provide an objective view of whether a country had met the criteria for certification [6]. The MECP additionally provides technical advice to the WHO on the certification criteria and procedures.

### Technical assistance, guidance, tools and training

During the E-2020 initiative, technical assistance from the WHO took several forms, including strengthening national strategic plans, facilitating and advising malaria programme reviews, leading trainings and workshops and providing technical advice to and assessments of standard operating procedures and guidelines. The staff of WHO also advised countries on development of operational research protocols to improve the evidence base for elimination strategies.

As a key component of the WHO’s normative function, updates to existing guidance and creation of new documents, tools and training materials were central to the E-2020 initiative; a list of key guidance documents and tools developed during the initiative, and evidence review groups held to explore a specific topic, are provided (Table 2). The need for different types of guidance was identified through frequent interaction with NMPs and other partners working on malaria elimination, while lessons learned from malaria-eliminating countries were used to improve and refine the content of guidance documents and tools.

**Table 2:** Guidance, tools and training material developed by WHO to support countries to eliminate malaria, achieve certification and prevent re-establishment

Technical product	Purpose	Description
*A framework for malaria elimination* (2017) [24]	Provide malaria-endemic countries with advice on the tools, activities and dynamic strategies required to achieve interruption of transmission and prevention of re-establishment	A guidance document that should serve as the basis for national malaria elimination plans after adaptation to the local context
Malaria Elimination Audit Tool (available by request from malaria-elimination@who.int)	Assist countries to translate the guidance in *A framework for malaria elimination* into programmatic activities and audit their performance	Identifies the key components of a malaria elimination or prevention of re-establishment programme and prompts programmes to evaluate their level of implementation, identify gaps and weaknesses and develop recommendations to strengthen programmes
Evidence review on border malaria [25]	Better define the concept of border malaria and identify the factors that might influence transmission in border areas	Defines border malaria and provides several case studies that explore different typologies of situations along the border
Evidence review on malariogenic potential [26]	Improve definitions of concepts, review available methodologies for assessing the components and advise on approaches to measuring malariogenic potential	Reviews approaches to measurement of the components of malariogenic potential and provides case studies from several countries
*Malaria surveillance, monitoring and evaluation: a reference manual* (2018) [27]	Serve as a reference document for guidance on strengthening malaria surveillance systems, including for elimination, with important chapters on entomologic surveillance and surveillance of drug efficacy in low transmission settings	A reference document for guidance on strengthening malaria surveillance systems that provides information to develop national standard operating procedures for case surveillance, drug efficacy monitoring and entomological surveillance in elimination settings
Malaria elimination surveillance assessment (available by request from malaria-elimination@who.int)	Assess the performance, quality, completeness and output of surveillance systems in elimination settings	Provides a framework for evaluation of case-based surveillance and response in elimination settings and a set of spreadsheet tools for assessment of data quality and completeness
*Preparing for certification of malaria elimination * [12]	Guide countries to prepare for certification, including a template for the national malaria elimination report	A manual to help countries understand the criteria for certification and the process that will be followed after certification is requested
Elimination training curriculum (available by request from malaria-elimination@who.int)	Facilitate understanding and implementation of the new elimination and surveillance guidance from WHO	14 modules and one tabletop exercise covering all aspects of elimination of malaria, based on WHO guidance

In 2019, using the model developed for the Stop Transmission of Polio (STOP) programme, the WHO piloted a new approach to provide technical and operational support to eliminate the country’s last foci of malaria transmission [7]. Field-experienced public health practitioners with malaria experience were recruited from other malaria-endemic countries and matched with a district or province in an eliminating country. After completion of elimination training, STOP-malaria consultants spent up to two 11-month assignments working at the subnational level with district authorities to support case management, surveillance and vector control for elimination. The pilot of the STOP-malaria programme in 2019 included three consultants; in 2020, the programme was expanded to five countries.

### Networking and reviews of national malaria elimination programmes

Convening countries to share their insights, innovations, challenges and best practices with other countries across the globe was effective in stimulating countries to try new approaches and creating a collective sense of shared goals. The WHO sponsored three global fora of malaria-eliminating countries (2–3 day meetings) during the E-2020 initiative [8–10]. At each meeting, NMPs presented on the strategies they were employing and their progress towards elimination. The agenda for each meeting contained a mixture of technical presentations on new guidance developed by the WHO and lessons learned from countries working towards elimination. The MEOC participated in two meetings, interacting directly with programme staff, reviewing their progress and providing recommendations to the WHO and the programme representatives on how to accelerate to elimination.

In 2019, the WHO, with advice from the MEOC, selected seven countries considered to be on track to eliminate malaria by 2020 (i.e., fewer than 100 indigenous cases in the year prior) to participate in a focused review of their programme’s performance and achievements [11]. NMPs presented an in-depth assessment of their strengths and weaknesses and worked with MEOC experts to identify solutions to programmatic bottlenecks. An important challenge common to most programmes was the need to improve cross-border collaboration to reduce malaria transmission in border areas, particularly along borders with higher-burden countries. A second focused review of countries considered off track to meet the 2020 elimination goal was planned for March 2020, but this meeting was canceled due to concerns over the spread of the SARS-COV2 virus.

### Certification and prevention of re-establishment

Countries that have interrupted malaria transmission and report zero indigenous cases for at least 3 consecutive years (defined as 36 months) are eligible to request WHO certification of their malaria-free status [12]. The two criteria for certification are: proof, beyond a reasonable doubt, that local transmission of all human malaria parasites has been fully interrupted; and evidence that an adequate programme for preventing re-establishment of transmission is fully functional throughout the country. Fulfilling this latter criterion may require integration of aspects of the malaria elimination programme that operated somewhat or completely independently of the general health services along with additional considerations such as traveler’s health, cross-border collaboration and involvement of sectors such as tourism and education to promote prevention of re-establishment.

To receive WHO certification, countries must prepare a national elimination report to explain the strategies and activities used to achieve elimination, to present proof that no indigenous malaria cases have occurred for at least the past 36 months and to summarize the country’s strategy to prevent re-establishment of transmission. The MECP conducts an in-country certification evaluation mission to verify the findings in the national elimination report and based on its findings, recommends to the WHO Director-General whether the country should be granted certification at present or some future time.

After certification, countries are included in the *Register of areas where malaria elimination has been achieved*, the official WHO list of certified countries [13]. Countries continue to report malaria cases annually to the WHO to monitor for potential re-establishment of transmission, which could result in loss of certification.

### Monitoring the impact of COVID-19 on the E-2020 countries

In March 2020 after many countries began implementing severe movement restrictions to contain the COVID-19 pandemic, the WHO instituted several efforts to help countries respond to the crisis while maintaining essential services, including malaria elimination. The GMP created six cross-partner workstreams to address the challenges posed by COVID-19 to malaria, including groups working on surveillance, supplies and commodities and efforts to mitigate the COVID pandemic on malaria. The WHO issued specific guidance on malaria interventions during the pandemic [14]. Regional elimination focal points instituted weekly or monthly calls with the WHO offices in malaria-eliminating countries to monitor the extent of disruption caused by the pandemic and to identify any countries in critical need of medications or supplies to avoid service interruption.

### Evaluating outcomes among the E-2020 countries

The number of indigenous malaria cases in the E-2020 countries was compared between 2020 and 2016, which was considered as the baseline year since the initiative was launched in 2017. To understand how baseline characteristics compared between E-2020 and other malaria-endemic countries, basic health and economic indicators for 2016 were extracted from the WHO’s Global Health Observatory and the World Bank’s World Development Indicators for the 90 countries considered malaria-endemic at baseline, and Paraguay [15, 16].

The number of indigenous malaria cases reported from the E-2020 countries between 2016 and 2019 was obtained from the *World Malaria Report 2020*; data for 2020 were obtained directly from NMPs [17]. Nonparametric statistics were used to describe the number of indigenous cases across the E-2020 countries at baseline and changes from 2016 to 2020 and scatter plots to visually compare the association between health and development indicators and absolute and relative changes in indigenous case numbers over the period of the initiative.

## Results

In 2016, 14 of the 21 (67%) E-2020 countries were classified as ‘upper middle’ or ‘high’ income according to their gross domestic product per capita (GDPpc; Table 3). Compared to the 70 other malaria-endemic countries in 2016, the E-2020 countries had a higher GDPpc, lower under-five mortality rate (U5MR), higher universal health care index of service coverage (UHC index), higher rates of literacy and greater access to electricity. These trends held true within each region except for South-East Asia.

**Table 3:** Key health and development indicators at baseline for the E-2020 countries and comparisons with other malaria-endemic countries [15, 16]

WHO region/country	Gross domestic product per capita (USD)^a^	Income classification^b^	Under-five mortality rate^c^	Universal health care index of service coverage^d^	Literacy rate (%)^e^	Access to electricity (%)^f^
Region of the Americas						
Belize	$4818	Upper middle	14.5	66	77	97
Costa Rica	$11,667	Upper middle	8.9	76	98	100
Ecuador	$6060	Upper middle	14.8	76	94	99
El Salvador	$3806	Lower middle	14.7	75	88	96
Mexico	$8745	Upper middle	15.7	76	95	100
Paraguay	$5319	Upper middle	21.5	68	95	98
Suriname	$5539	Upper middle	19.7	70	94	96
E-2020 countries (n = 7)	$6565		15.7	72	94	98
Malaria-endemic countries (n = 12)	$5563		24.9	70	90	91
Region of Africa						
Algeria	$3946	Upper middle	24.8	76	81	100
Botswana	$7244	Upper middle	43.6	61	87	60
Cabo Verde	$3131	Lower middle	18.1	67	87	89
Comoros	$1273	Lower middle	69.5	49	59	77
Eswatini	$3426	Lower middle	58.9	60	88	70
South Africa	$5273	Upper middle	36.2	69	87	84
E-2020 countries (n = 6)	$4049		41.9	64	82	80
Malaria-endemic countries (n = 38)	$1546		75.3	42	62	40
Region of the Eastern Mediterranean						
Iran, Islamic Republic of	$5253	Upper middle	15.5	70	86	100
Saudi Arabia	$19,879	High	7.9	73	95	100
E-2020 countries (n = 2)	$12,566		11.7	72	90	100
Malaria-endemic countries (n = 6)	$1424		75.7	37	54	64
Region of South-East Asia						
Bhutan	$2931	Lower middle	31.8	59	67	100
Nepal	$777	Low	34.6	51	68	91
Timor-Leste	$1354	Lower middle	49.0	49	68	77
E-2020 countries (n = 3)	$1687		38.5	53	68	89
Malaria-endemic countries (n = 6)	$2792		27.4	60	84	80
Region of the Western Pacific						
China	$8148	Upper middle	9.9	76	97	100
Korea, Rep. of	$29,289	High	3.4	85		100
Malaysia	$9818	Upper middle	8.2	71	95	100
E-2020 countries (n = 3)	$15,752		7.2	77	96	100
Malaria-endemic countries (n = 7)	$2353		33.0	53	89	75
Global						
E-2020 countries (n = 21)	$7033		24.8	68	86	92
Malaria-endemic countries (n = 70)^g^	$2388		58.0	49	71	58

^a^Gross domestic product in $US divided by the midyear population

^b^World Bank classification for 2016 based on gross national income per capita in 2015

^c^Number of deaths per 1000 live births

^d^Reported on a unitless scale of 0 to 100 and computed as the geometric mean of 14 tracer indicators of health service coverage. As no UHC index was published for 2016, the values for 2015 were used

^e^Proportion of people aged 15 and above who can both read and write with understanding a short simple statement about their everyday life

^f^Proportion of population with access to electricity

^g^Includes one malaria-endemic country (Tajikistan) in Europe

Before the start of the E-2020 initiative, the malaria-endemic E-2020 countries (excluding Paraguay, which was reclassified in 2017 as non-endemic at the start of the E-2020) reported a median of 165.5 indigenous cases (interquartile range [IQR] 14.25–563.75) (Table 4); three countries reported more than 1000 cases and four reported fewer than 10 cases. In 2020, the E-2020 countries reported a median of 78 indigenous cases (IQR 0–356), with three countries reporting more than 1000 cases and eight reporting fewer than 10 cases.

**Table 4:** Number of indigenous malaria cases by country, 2016–2020 [17]

WHO region/country	2016	2017	2018	2019	2020	Absolute difference 2020–2016	Relative difference 2020–2016
Americas							
Belize	4	7	3	0	0	− 4	− 100%
Costa Rica	4	12	70	95	90	86	2150%
Ecuador	1191	1275	1653	1803	1934	743	62%
El Salvador	12	0	0	0	0	− 12	− 100%
Mexico	551	736	803	618	356	− 195	− 35%
Paraguay^a^	0	0	0	0	0	0	
Suriname	76	19	29	95	147	71	93%
Africa							
Algeria	0	0	0	0	0	0	
Botswana	659	1847	534	169	884	225	34%
Cabo Verde	48	423	2	0	0	− 48	− 100%
Comoros	1467	3896	15,613	17,599	4546	3079	210%
Eswatini	250	440	686	239	233	− 17	− 7%
South Africa	4323	23,381	9540	3096	4463	140	3%
Eastern Mediterranean							
Iran, Islamic Republic of	81	60	0	0	0	− 80	− 100%
Saudi Arabia	272	177	61	38	83	− 189	− 69%
South-East Asia							
Bhutan	15	11	6	2	22	7	47%
Nepal	507	623	493	127	73	− 434	− 86%
Timor-Leste	81	16	0	0	3	− 78	− 96%
Western Pacific							
China	1	0	0	0	0	− 1	− 100%
Korea, Rep. of	602	436	501	485	356	− 246	− 41%
Malaysia	266	85	0	0	0	− 266	− 100%
All E-2020 countries							
Median^b^	165.5	131	45	66.5	78	− 7	− 41%

^a^Two E-2020 countries (Algeria and Paraguay) finalized classification of several cases after selection for the initiative was complete and determined that they had actually achieved their first year of zero indigenous malaria cases in 2014 and 2012, respectively; Paraguay was, therefore, classified as malaria-free in 2015 while Algeria was still malaria-endemic as it had yet to complete 3 years malaria-free

^b^Paraguay is not included in the calculation of the median because it was no longer classified as malaria-endemic in 2016

**Fig. 1:**
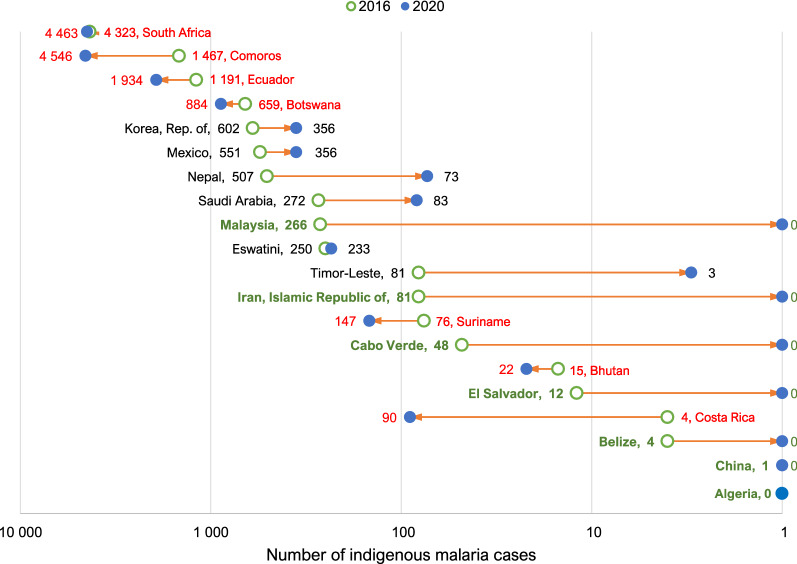
Change in number of indigenous cases among the E-2020 countries between 2016 and 2020. Paraguay was excluded as it was reclassified as non-endemic in 2016. Countries with increases, decreases or no change in numbers of indigenous malaria cases over the period 2016–2020 are indicated in red, green and black, respectively

The majority (60%, 12/20) of E-2020 countries reported reductions in absolute numbers of indigenous cases between 2016 and 2020 that ranged from 1 to 434 fewer cases and relative declines of 7% to 100% (Fig. 1); seven countries (Algeria, Belize, Iran [Islamic Republic of], Cabo Verde, China, El Salvador, and Malaysia) reported at least 1 year of zero indigenous cases and maintained their malaria-free status through the end of 2020. The likelihood of interrupting transmission and maintaining a malaria-free status through 2020 was associated with the number of indigenous malaria cases reported in 2016: while 75% of the countries reporting 0–9 cases in 2016 interrupted transmission and maintained that status through 2020, the proportion declined to 50% of countries with 10–99 cases, 14% of countries with 100–999 cases and 0% of countries with 1000 or more cases at baseline. All seven countries that interrupted transmission and maintained zero indigenous cases through 2020 reported fewer than 300 cases in 2016; among the other six countries with fewer than 300 cases at baseline, one (Timor-Leste) reached zero indigenous cases for 2 years (2018–2019) before experiencing an outbreak of local malaria transmission in 2020; two (Eswatini and Saudi Arabia) reported declines in cases but did not reach elimination; and three (Bhutan, Costa Rica and Suriname) reported an increase in cases.

The seven countries (Botswana, Comoros, Costa Rica, Ecuador, Mexico, South Africa and Suriname) that experienced increases in indigenous malaria cases over the period of the initiative reported between 7 and 3079 more cases, and relative increases from 3% to more than 2000%. No clear associations between health and development indicators and either absolute or relative change in numbers of cases among the E-2020 countries were identified through visual examination of scatter plots (Additional file 1: Appendices S1, S2).

E-2020 countries faced significant challenges due to the COVID-19 pandemic, including redeployment of NMP staff to assist with the pandemic response, movement restrictions that prevented case investigations from being conducted in person and reductions in care-seeking behavior due to patient mistrust or fear. All but one of the E-2020 countries reported reductions in imported malaria cases during 2020 because of movement and border restrictions [18]. Two E-2020 countries, Bhutan and Timor-Leste, experienced outbreaks of malaria associated with the COVID-19 pandemic. In Bhutan, delays in delivery of malaria prevention interventions attributed to the pandemic resulted in several malaria outbreaks and indigenous transmission [19]. Movement restrictions in Timor-Leste delayed responses to imported cases along the border with Indonesia, leading to an outbreak and indigenous transmission (M. Mota, personal communication). The other E-2020 countries found ways to adapt to the restrictions necessitated by the pandemic and largely mitigated the impact of the pandemic on malaria elimination progress.

Algeria and Paraguay were certified malaria-free by the WHO in 2019 and 2018, respectively [20, 21]. China and El Salvador reached 3 years of zero indigenous cases in 2020, and both were certified malaria-free by the WHO in 2021 [22, 23]. The other E-2020 countries that have interrupted malaria transmission (Belize, Iran [Islamic Republic of], Cabo Verde and Malaysia) are either currently eligible to request WHO certification or will become eligible by the end of 2021.

## Discussion

The 194 member-states of the WHO came to the World Health Assembly in 2015 and committed to achieving elimination in 35 malaria-endemic countries by 2030. The E-2020 initiative was formed by the WHO to help countries achieve their malaria elimination goals and thereby contribute to the 2020 GTS elimination milestone. Among the 20 E-2020 countries considered malaria-endemic in 2015, seven (Algeria, Belize, Cabo Verde, China, El Salvador, Iran [Islamic Republic of] and Malaysia) interrupted malaria transmission and maintained their malaria-free status through 2020. These seven countries contributed to the achievement of the 2020 GTS elimination milestone, along with three others (Azerbaijan, Sri Lanka and Tajikistan) that were malaria-endemic in 2015 but achieved interruption of transmission before the launch of the E-2020 in 2017.

All seven E-2020 countries that achieved elimination and maintained their malaria-free status through 2020 reported fewer than 300 indigenous cases in 2016; however, out of the 13 countries with fewer than 300 cases at baseline, six did not achieve elimination by 2020, illustrating the difficulties faced by countries working to interrupt malaria transmission even when numbers of malaria cases are already extremely low. Although E-2020 countries had better health and development indicators than other malaria-endemic countries not invited to participate in the initiative, within the E-2020 initiative there was no association between these indicators and interruption of transmission. It is likely that many factors that are difficult to measure, including political will, leadership and management, adequate human, material and financial resources, health system performance and level of access to health services for all who need them, are important in determining whether a country achieves interruption of transmission.

The analysis of changes in indigenous case numbers experienced by the E-2020 countries is presented in this report to illustrate the experiences of the countries over the period of the initiative and is not meant to imply causality. An evaluation of the direct impact of the E-2020 initiative is prevented by the absence of experimental design, purposive selection of countries and lack of controls. Additionally, the WHO’s support to the E-2020 countries was complemented by efforts from other organizations. The Global Fund to Fight AIDS, Tuberculosis and Malaria, the Gates Foundation, the Inter-American Development Bank and other donors provided direct financial support to many elimination programmes as well as technical support through partners such as the University of California at San Francisco and the Clinton Health Access Initiative. Most of the E-2020 countries are also members of regional networking initiatives such as the Asia Pacific Malaria Elimination Network, the Elimination Eight Initiative (southern Africa) and the Regional Malaria Elimination Initiative (Meso-America). Finally, the E-2020 countries themselves possess significant technical expertise from within their NMPs, academic institutions and civil society organizations that they have drawn on to strengthen their elimination strategies.

The current COVID-19 pandemic has increased the complexity of the elimination effort by diverting human and financial resources, limiting the mobility of technical teams, disrupting healthcare seeking behavior and restricting the movement of technical partners. Two countries among the E-2020 likely missed their elimination goals because of the pandemic. However, NMPs continue to adapt to changing circumstances and most countries have benefitted from reductions in importation of malaria cases during the pandemic period. The overall impact of the COVID-19 pandemic on elimination and prevention of reestablishment may not be known for some time, but countries continue to maintain essential services during this difficult period to prevent severe disruption of progress towards their malaria elimination goals.

## Conclusions

Malaria elimination must be driven and owned by national governments as only countries themselves are able to galvanize the necessary political will and domestic financing that is essential for elimination to be achieved and re-establishment of transmission prevented. The WHO serves as the final authority to grant malaria-free certification and, therefore, plays a critical role in supporting and guiding countries to elimination. The E-2020 initiative was the first effort by the WHO since the end of the Global Malaria Eradication Programme to assist a large group of countries to move more quickly to elimination. The outcomes for countries in the initiative were mixed: although seven countries interrupted transmission and four were certified malaria-free, 13 countries did not achieve their elimination goals. However, the body of lessons learned from the countries participating in the initiative has helped the WHO sharpen and clarify its elimination guidance and develop new tools to assist countries to assess and strengthen their elimination programmes and strategies to prevent re-establishment.

The certification of four E-2020 countries has been helpful in generating positive news coverage for malaria during a period when overall progress has stalled. Maintaining a high level of political support for malaria is critical for all countries but particularly for those nearing elimination when the burden of malaria is very low and other public health priorities take prominence. The international and domestic press coverage of each WHO certification of elimination reinforces the importance of the goal and helps generate momentum for other countries approaching elimination.

The achievements of the E-2020 initiative remain to be consolidated: countries that interrupted transmission must be supported to maintain their malaria-free status and gain WHO certification; and those that have achieved certification must be reinforced to prevent re-establishment of transmission. However, the 2025 GTS milestone is now in sight, and the global goal of 10 more countries eliminating malaria must be met. Building on the strategies, accomplishments and lessons learned of the E-2020 initiative, the WHO launched the E-2025 initiative in 2021 with 25 countries seeking to interrupt malaria transmission by 2025 [18].

## Supplementary Information

**Additional file 1: Appendix S1.** Association between absolute reductions in indigenous malaria cases between 2016 and 2020 and development and health indicators among E-2020 countries. **Appendix S2.** Association between relative reductions in indigenous malaria cases between 2016 and 2020 and development and health indicators among E-2020 countries.

## Data Availability

The datasets used and analysed during the study period are available in Additional file.

## Abbreviations

E-2020: Elimination 2020
GDPpc: Gross domestic product per capita
GMP: Global Malaria Programme
GTS: Global technical strategy for malaria 2016–2030
IQR: Interquartile range
MEAT: Malaria Elimination Audit Tool
MECP: Malaria Elimination Certification Panel
MEOC: Malaria Elimination Oversight Committee
NMP: National malaria programme
STOP: Stop Transmission of Polio
UHC: Universal health coverage
U5MR: Under-five mortality rate
WHO: World Health Organization
