# Supplementary material for: Supporting countries to achieve their malaria elimination goals: the WHO E-2020 initiative
Source: Malar J. 2021 Dec 20;20:481. doi: 10.1186/s12936-021-03998-3 (PMC8686104; doi:10.1186/s12936-021-03998-3)
Supplement: Supplementary file 1 — Additional file 1: Appendix S1. Association between absolute reductions in indigenous malaria cases between 2016 and 2020 and development and health indicators among E-2020 countries. Appendix S2. Association between relative reductions in indigenous malaria cases between 2016 and 2020 and development and health indicators among E-2020 countries. [file 12936_2021_3998_MOESM1_ESM.pdf]

Appendix

Appendix S1. Association between absolute reductions in indigenous malaria cases between 2016 and 2020 and development and health indicators among E-2020 countries.*

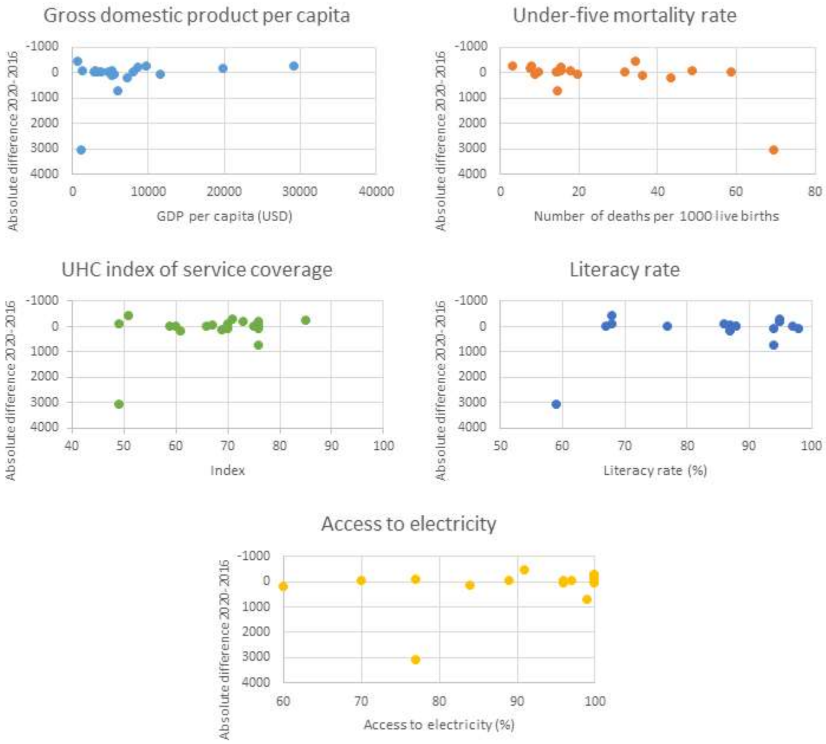

*Two E-2020 countries (Algeria and Paraguay) were excluded as both reported 0 indigenous cases in 2016 and 2020.

Appendix S2. Association between relative reductions in indigenous malaria cases between 2016 and 2020 and development and health indicators among E-2020 countries.*

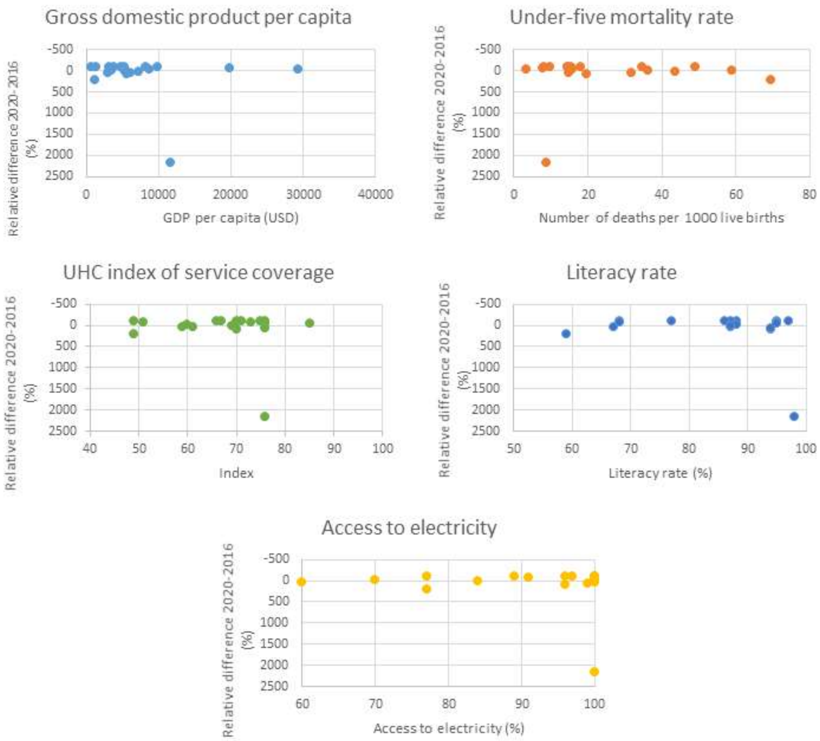

*Two E-2020 countries (Algeria and Paraguay) were excluded as both reported 0 indigenous cases in 2016 and 2020.
